# Internet Use for Health-Related Information via Personal Computers and Cell Phones in Japan: A Cross-Sectional Population-Based Survey

**DOI:** 10.2196/jmir.1796

**Published:** 2011-12-14

**Authors:** Yoshimitsu Takahashi, Tomoko Ohura, Tatsuro Ishizaki, Shigeru Okamoto, Kenji Miki, Mariko Naito, Rie Akamatsu, Hiroki Sugimori, Nobuo Yoshiike, Koichi Miyaki, Takuro Shimbo, Takeo Nakayama

**Affiliations:** ^1^Department of Health InformaticsKyoto University School of Public HealthKyotoJapan; ^2^Faculty of Care and RehabilitationSeijoh UniversityTokai, AichiJapan; ^3^Human Care Research TeamTokyo Metropolitan Institute of GerontologyTokyoJapan; ^4^Regional Research InstituteOsaka University of Economics and LawYao, OsakaJapan; ^5^Department of Preventive MedicineGraduate School of MedicineNagoya UniversityNagoya, AichiJapan; ^6^Graduate School of Humanities and SciencesOchanomizu UniversityTokyoJapan; ^7^Department of Preventive MedicineGraduate School of Sports and Health SciencesDaito Bunka UniversityHigashimatsuyama, SaitamaJapan; ^8^Community NutritionFaculty of Health SciencesAomori University of Health and WelfareAomoriJapan; ^9^Department of Clinical Research and InformaticsNational Center for Global Health and MedicineTokyoJapan

**Keywords:** eHealth, email, cell phones, health literacy, information-seeking behavior, patient-provider communication

## Abstract

**Background:**

The Internet is known to be used for health purposes by the general public all over the world. However, little is known about the use of, attitudes toward, and activities regarding eHealth among the Japanese population.

**Objectives:**

This study aimed to measure the prevalence of Internet use for health-related information compared with other sources, and to examine the effects on user knowledge, attitudes, and activities with regard to Internet use for health-related information in Japan. We examined the extent of use via personal computers and cell phones.

**Methods:**

We conducted a cross-sectional survey of a quasi-representative sample (N = 1200) of the Japanese general population aged 15–79 years in September 2007. The main outcome measures were (1) self-reported rates of Internet use in the past year to acquire health-related information and to contact health professionals, family, friends, and peers specifically for health-related purposes, and (2) perceived effects of Internet use on health care.

**Results:**

The prevalence of Internet use via personal computer for acquiring health-related information was 23.8% (286/1200) among those surveyed, whereas the prevalence via cell phone was 6% (77). Internet use via both personal computer and cell phone for communicating with health professionals, family, friends, or peers was not common. The Internet was used via personal computer for acquiring health-related information primarily by younger people, people with higher education levels, and people with higher household incomes. The majority of those who used the Internet for health care purposes responded that the Internet improved their knowledge or affected their lifestyle attitude, and that they felt confident in the health-related information they obtained from the Internet. However, less than one-quarter thought it improved their ability to manage their health or affected their health-related activities.

**Conclusions:**

Japanese moderately used the Internet via personal computers for health purposes, and rarely used the Internet via cell phones. Older people, people with lower education levels, and people with lower household incomes were less likely to access the Internet via cell phone. The Internet moderately improved users’ health-related knowledge and attitudes but seldom changed their health-related abilities and activities. To encourage communication between health providers and consumers, it is important to improve eHealth literacy, especially in middle-aged people. It is also important to make adequate amendments to the reimbursement payment system and nationwide eHealth privacy and security framework, and to develop a collaborative relationship among industry, government, and academia.

## Introduction

The number of Internet users has increased considerably worldwide [1]. The Internet is used for health purposes by the general public, and the importance of the Internet as a source of health information is growing [2-5]. The term eHealth refers to health services and information delivered or enhanced through the Internet [1,6-8]. To monitor health consumers’ use, attitudes, and activities regarding health-related information and eHealth, national representative surveys were conducted in the United States (Health Information National Trends Survey by the National Cancer Institute) [3,9-11] and Europe (eHealth Consumer Trends Survey funded by the European Commission) [5,12,13]. These surveys revealed an increase in the prevalence of Internet use for health-related information among the general public. The prevalence in the United States was approximately 20% in 2001 [2] and 40% in 2003 [3]; the prevalence in Europe was 42% in 2005 and 52% in 2007 [5]. Several studies, however, showed that people still valued and used more conventional sources of health-related information, including health professionals, family, television, and newspapers, although the conventional sources decreased in importance [14,15]. It was also shown that the effects of Internet use on health-related attitudes and activities, such as active communication and actual health care utilization, have not yet been substantial [2,5].

 A 2007 Japanese national survey showed that 69% used the Internet in the past year, 61% through personal computers and 57% through cell phones [16]. Japan’s cell phones are technologically enhanced and divergent from globalization, a phenomenon labeled the Galápagos syndrome [17]. They are ready for the Internet and email, have high-resolution cameras, receive television programs, and can be used as credit cards and boarding passes. Even the average person can have an advanced cell phone, so many Japanese rely on their cell phones rather than personal computers for Internet access [17]. However, little is known about the use of, attitudes toward, and activities regarding eHealth in the Japanese population. Clearer fundamental information is required as a foundation for discussing the role of the Internet in health care. It is assumed that changes in the information technology environment have affected Internet use for health-related information in several ways in Japan, as well as in the United States and Europe, where Web 2.0 has been changing the way medical information is handled (eg, personal health records) [18,19]. Therefore, we hypothesized that the prevalence and effects of Internet use for health-related information in Japan are similar to those in the United States and Europe. Moreover, since many Japanese rely on their cell phones for Internet access, we think that Internet use via cell phone can be as effective as Internet use via personal computer [16,17,20].

This study aimed to measure the prevalence of Internet use for health-related information compared with other sources, to examine user characteristics, and to examine the association of Internet use with user knowledge, attitudes, and activities regarding health-related information in Japan. Additionally, we examined the extent of Internet use via personal computers and cell phones.

## Methods

### Study Design and Participants

We designed a cross-sectional survey of the Japanese general population aged 15–79 years. We used a scheduled omnibus survey conducted by Nippon Research Center Ltd [21], which included 1200 participants. Study participants were selected by proportional quota sampling to collect a nationally representative sample in Japan, and a self-reported questionnaire survey was performed in September 2007. In proportion to regions and city sizes, 200 areas were proportionately selected corresponding to the stratification of all nine regions (Hokkaido, Tohoku, Kanto, Hokuriku, Tokai, Kinki, Chugoku, Shikoku, and Kyushu) and five city sizes (15 large cities: Sapporo, Sendai, Saitama, Chiba, Tokyo, Kawasaki, Yokohama, Shizuoka, Nagoya, Kyoto, Osaka, Kobe, Hiroshima, Kitakyushu, and Fukuoka; cities with over 150,000 people; cities with over 50,000 people; cities with fewer than 50,000 people; and nonurban areas). Households were randomly selected from a database of house maps. Individuals were allocated to reflect the area’s stratification by sex, age, and job status. Interviewers visited selected households, requested that individuals fill out questionnaires, and collected questionnaires completed by allocated individuals a few days later. In the case that interviewers could not collect a questionnaire from a target participant, interviewers visited the next target participant that reflected the area’s demographics. Sampling continued until we had 1200 completed questionnaires. All respondents were provided ¥1000 (about US $10 at the time of writing) as payment on completion of the questionnaire.

### Measurements

The survey contained a set of questions about participant characteristics, use of the Internet for health-related information, and the perceived effects of Internet use on knowledge, attitudes, and activities for health purposes. Almost all items were derived from the original questionnaire used in Baker and colleague’s study [2]. We added some original items regarding cell phones.

We collected basic demographic data from participants, including age, sex, household income, level of education, and place of residence. Health-related characteristics were self-reported health status (excellent, very good, good, fair, or poor) and chronic diseases: hypertension, diabetes or hyperglycemia, cancer, heart problems (heart attack, angina due to coronary heart disease, heart failure, or other heart problems), depression, obesity, and hyperlipidemia. The main outcome of this study was frequency of Internet use for any purpose and ownership of cell phones.

We classified Internet use into four types: (1) use of a Web browser via personal computer, (2) use of a Web browser via cell phone, (3) use of email via personal computer, and (4) use of email via cell phone. We prepared four questions: “How often do you use a Web browser (or email) to acquire information or advice for health care via your personal computer (or through your cell phone)?” We defined “Internet use” as more than once a year. In addition, to compare the extent of Internet use, we also measured the extent other sources were used for health-related information (television, newspapers, radio, magazines, direct mail, and public relations magazines). To investigate the extent of interactive Internet use for health-related communication, we asked participants about their use of the Internet for three purposes: “to contact doctors or other health care providers,” “to contact a family member or friend about health or health care,” and “to contact other people who have similar health conditions or concerns.” We examined the extent of use for these three purposes via personal computer and cell phone.

 We examined the perceived effects of Internet use on knowledge and attitudes using participant responses (strongly agree, agree, disagree, or strongly disagree) to the following statements: “improved my understanding of symptoms, conditions, or treatments in which I was interested,” “improved my ability to manage my health care needs without visiting a doctor or other health care provider,” “led me to seek care from different doctors or health providers than I otherwise would have,” and “affected the way I eat or exercise.” We also examined Internet user confidence or anxiety (“I felt confident,” “I wasn’t influenced,” “I felt anxious,” or “I’ve never obtained [this information]” after obtaining the following health-related information: “information on diseases you have,” “information on diseases you want to prevent,” “information on treatment of diseases,” “information on doctors and health care facilities,” “information on peers,” and “information on a healthy lifestyle, fitness, or nutrition.”

We examined the perceived effects of Internet use on activities by collecting data on the number of times participants visited a health professional and the number of times they telephoned them. Additionally, we asked “Have you ever told health professionals about information from the Internet?”

### Statistical Analysis

We tabulated the responses and computed the prevalences. Then, we used logistic regression analysis to investigate the relationships between Internet use for health-related information and respondent characteristics (age, sex, annual household income, level of education, place of residence, and self-reported health status). We evaluated eight logistic regression models. The outcomes for models 1–4 were use of the Internet via personal computer for (1) acquiring health-related information, (2) contacting health professionals, (3) contacting family/friends about health-related information, and (4) contacting peers. The outcomes for models 5–8 were use of the Internet via cell phone for (5) acquiring health-related information, (6) contacting health professionals, (7) contacting family/friends, and (8) contacting peers. For each variable, we report odds ratios and 95% confidence intervals. The Hosmer-Lemeshow goodness-of-fit test was performed for each model. All analyses were performed using SPSS version 18.0 (IBM Corporation, Somers, NY, USA). All *P* values were 2-sided, with *P* < .05 considered statistically significant.

### Ethical Considerations

The purpose of the study was explained on the first page of the questionnaire, and we declared that responses to questionnaires were regarded as informed consent. This survey was conducted as an unlinked anonymous survey. The study protocol was approved by the Ethics Committee of Kyoto University Faculty of Medicine.

## Results

### Participant Characteristics

Characteristics of the 1200 survey participants included in our analysis are shown in Table 1. The mean (SD) age was 46.4 (17.4) years, 49.6% (595/1200) of the participants were male, 18.7% (224) had at least a college education, and 35.6% (426) had a household income of ¥6,000,000 (about US $60,000) or more. “Poor” general health was reported by 7% (82) of respondents and 31.4% (377) had at least one chronic condition. In addition, 41.5% (498) had used the Internet more than once a week for general purposes; 81.3% (975) had cell phones, and the prevalence of Internet use via cell phone was 41.8% (502).

**Table 1:** Participant characteristics (N = 1200)

		n	%
**Age (years)**			
	15–19	75	6
	20–34	285	23.8
	35–49	295	24.6
	50–64	324	27.0
	65–74	169	14.1
	75–79	52	4
	Mean (SD)	46.4 (17.4)	
**Sex (male)**		595	49.6
**Household income (¥1000) ^a^**			
	0–2999	194	16.2
	3000–5999	418	34.8
	6000–9999	314	26.2
	10,000–	112	9.3
	Unknown	162	13.5
**Education (years)**			
	0–12	728	60.7
	13–15	241	20.1
	16–	224	18.7
	Unknown	7	1
**Health status**			
	Excellent/very good	248	20.7
	Good	350	29.2
	Fair	520	43.3
	Poor	82	7
**Place of residence**			
	Urban^b^	690	57.5
	Nonurban	510	42.5
**Chronic conditions**			
	≥3	33	3
	2	86	7
	1	258	21.5
	0	823	68.6

^a^ ¥1000 = about US $10.

^b^ Cities with a population of at least 150,000 people.

### Prevalence of Using Various Sources for Acquiring Health-Related Information

Table 2 shows the prevalence of use for health-related information by source. We regarded *u* *se* as use at least once every year. The prevalence of Internet use (Web browser or email) via personal computers for acquiring health-related information was 23.8% (286), and 6% (77) for Internet use via cell phones. Television (60.1%, 721) and newspapers (50.3%, 604) were widely used. The prevalence was 7% (79) and 3% (36) for contact with health professionals (doctors or other health care providers), 8.6% (103) and 12.3% (148) for contact with family or friends, and 4% (52) and 6% (67) for contact with peers (other people with similar health conditions or concerns) for interactive use of the Internet via personal computer and cell phone, respectively.

**Table 2:** Prevalence and frequency of Internet use for health purposes (N = 1200)

			Frequency of use, % (n)					
In the past year, about how often did you			Total ever in the past year	More than once per week	About once per week	About once per month	Every 2–3 months	Less than every 2–3 months
**To acquire health-related information, use**								
	Television		60.1 (721)	21.7 (260)	14.8 (178)	10.8 (129)	5 (54)	8.3 (100)
	Newspapers		50.3 (604)	17.3 (207)	13.3 (159)	9.1 (109)	4 (48)	7 (81)
	Public relations magazines		40.3 (484)	1 (17)	2 (27)	20.6 (247)	6 (77)	9.7 (116)
	Magazines		34.2 (410)	3 (38)	4 (50)	12.3 (147)	7 (83)	8 (92)
	Radio		19.1 (229)	5 (56)	4 (52)	4 (50)	2 (26)	4 (45)
	Direct mail		16.5 (198)	1 (15)	2 (25)	6 (68)	4 (45)	4 (45)
	Web browser via...							
		Personal computer	23.7 (284)	4 (45)	4 (47)	6 (69)	6 (67)	5 (56)
		Cell phone	5 (63)	1 (14)	1 (8)	1 (15)	1 (8)	2 (18)
	Email via...							
		Personal computer	5 (61)	1 (15)	1 (8)	1 (15)	1 (8)	1 (15)
		Cell phone	4 (48)	1 (14)	0 (6)	1 (12)	0 (2)	1 (14)
	The Internet (Web browser or email) via...							
		Personal computer	23.8 (286)	4 (48)	4 (47)	6 (70)	5 (65)	5 (56)
		Cell phone	6 (77)	2 (21)	1 (7)	2 (19)	1 (7)	2 (23)
**To contact health professionals, use**								
	The Internet via...							
		Personal computer	7 (79)	1 (8)	1 (6)	2 (18)	1 (17)	3 (30)
		Cell phone	3 (36)	2 (7)	0 (2)	1 (6)	1 (6)	1 (15)
**To contact a family member or friend about health or health care,use**								
	The Internet via...							
		Personal computer	8.6 (103)	2 (20)	1 (16)	2 (20)	2 (19)	2 (28)
		Cell phone	12.3 (148)	2 (29)	2 (25)	3 (34)	1 (15)	4 (45)
**To contact peers about health or health care, use**								
	The Internet via...							
		Personal computer	4 (52)	1 (14)	0 (4)	1 (8)	1 (8)	2 (19)
		Cell phone	6 (67)	1 (8)	1 (10)	1 (17)	1 (10)	2 (22)

### Characteristics of People Using the Internet for Health-Related Information

Table 3 shows logistic regression analysis results of the relationships between Internet use via personal computer for each health purpose and participant characteristic. We observed that participants over 50 years of age were significantly less likely to use the Internet via personal computer for acquiring health-related information, while those with an income over ¥10,000,000 or with more than 12 years of education were more likely to acquire information this way. Table 4 shows results on the use of cell phones. Participants aged 35–64 years were less likely than younger participants to use the Internet via cell phone to obtain health-related information.

 We also considered interactive use of the Internet for health-related communication (see Table 3 and Table 4). Overall, there were few statistically significant differences in characteristics. Women and those reporting good health status were more likely to use the Internet interactively. As with using the Internet via personal computer for acquiring information, higher rates of interactive Internet use might be related to younger age, higher education levels, and higher household incomes. A lower rate of interactive Internet use via cell phone might be associated with older age and lower education levels, but was not associated with household income. All eight models were shown to be well calibrated using the Hosmer-Lemeshow test (each *P* value > .31).

**Table 3:** Results of logistic regression models for Internet use via personal computer for each health purpose by demographic characteristics (N = 1200)

		For acquiring information	For contacting professionals	For contacting family/friends	For contacting peers
**Number of users (%)**		286 (23.8%)	79 (7%)	103 (8.6%)	52 (4%)
**Age (years), OR (95% CI)^a^**					
	15–19	0.5 (0.2–1.0)	0.2 (0.0–1.3)	0.6 (0.2–1.7)	0.3 (0.0–2.5)
	20–34	Reference	Reference	Reference	Reference
	35–49	1.2 (0.8–1.7)	1.0 (0.6–1.7)	0.9 (0.5–1.4)	0.8 (0.4–1.6)
	50–64	0.6 (0.4–0.9)^b^	0.7 (0.4–1.4)	0.7 (0.4–1.2)	0.7 (0.3–1.5)
	65–79	0.2 (0.1–0.4)^b^	0.4 (0.2–1.1)	0.3 (0.1–0.8)^b^	0.3 (0.1–1.1)
**Sex (female), OR (95% CI)^a^**		1.0 (0.8–1.4)	1.8 (1.1–2.9)^b^	1.5 (0.9–2.3)	1.7 (0.9–3.2)
**Household income (¥1000)^c^, OR (95% CI)^a^**					
	0–2999	Reference	Reference	Reference	Reference
	3000–5999	1.6 (0.9–2.7)	1.2 (0.5–3.0)	1.1 (0.5–2.5)	1.2 (0.4–3.4)
	6000–9999	1.7 (1.0–2.9)	1.6 (0.7–4.0)	1.5 (0.7–3.3)	1.2 (0.4–3.6)
	10,000–	2.5 (1.3–4.8)^b^	1.1 (0.4–3.4)	1.6 (0.7–4.0)	2.3 (0.7–7.3)
**Education (years), OR (95% CI)^a^**					
	0–12	Reference	Reference	Reference	Reference
	13–15	1.8 (1.2–2.6)^b^	1.9 (1.0–3.3)^b^	1.7 (0.9–2.9)	1.7 (0.8–3.6)
	16–	4.8 (3.3–6.8)^b^	2.6 (1.4–4.7)^b^	3.8 (2.3–6.4)^b^	2.8 (1.4–5.8)^b^
**Urban residence^d^, OR (95% CI)^a^**		1.4 (0.9–2.1)	0.6 (0.2–1.4)	1.1 (0.6–2.0)	0.7 (0.3–1.8)
**Health status, OR (95% CI)^a^**					
	Excellent/very good	Reference	Reference	Reference	Reference
	Good	1.4 (0.9–2.1)	1.0 (0.5–1.8)	1.3 (0.8–2.3)	3.0 (1.2–7.7)^b^
	Fair	1.1 (0.7–1.6)	0.9 (0.5–1.6)	0.7 (0.4–1.2)	1.9 (0.7–4.9)
	Poor	1.8 (0.9–3.6)	0.6 (0.2–2.3)	1.1 (0.4–3.1)	1.4 (0.3–7.6)
**Test for goodness-of-fit^e^**		*P* = .39	*P* = .66	*P* = .99	*P* = .59

^a^ Odds ratio (95% confidence interval).

^b^ Confidence interval does not include 1.0.

^c^ ¥1000 = about US $10.

^d^ Population of at least 150,000 people.

^e^ Hosmer-Lemeshow goodness-of-fit test.

**Table 4:** Results of logistic regression models for Internet use via cell phone for each health purpose by demographic characteristics (N = 1200)

		For acquiring information	For contacting professionals	For contacting family/friends	For contacting peers
**Number of users (%)**		63 (5%)	36 (3%)	148 (12.3%)	67 (6%)
**Age (years), OR (95% CI)^a^**					
	15–19	0.6 (0.2–1.6)	0.9 (0.3–3.1)	0.6 (0.3–1.2)	0.6 (0.2–1.9)
	20–34	Reference	Reference	Reference	Reference
	35–49	0.5 (0.3–1.0)^b^	0.3 (0.1–0.8)^b^	0.5 (0.3–0.8)^b^	0.6 (0.4–1.2)
	50–64	0.2 (0.1–0.4)^b^	0.2 (0.1–0.6)^b^	0.2 (0.1–0.3)^b^	0.2 (0.1–0.5)^b^
	65–79	n/a^c^	n/a^c^	0.0 (0.0–0.1)^b^	0.0 (0.0–0.3)^b^
**Sex (female), OR (95% CI)^a^**		1.2 (0.7–2.0)	1.3 (0.6–2.7)	2.0 (1.4–3.0)^b^	2.2 (1.3–3.9)^b^
**Household income (¥1000)^d^, OR (95% CI)^a^**					
	0–2999	Reference	Reference	Reference	Reference
	3000–5999	1.2 (0.5–3.1)	0.6 (0.2–1.9)	0.7 (0.4–1.4)	1.0 (0.4–2.4)
	6000–9999	1.2 (0.4–3.2)	1.2 (0.4–3.5)	1.1 (0.6–2.0)	0.9 (0.4–2.4)
	10,000–	1.7 (0.6–5.4)	0.7 (0.2–3.3)	1.0 (0.5–2.2)	1.0 (0.3–3.2)
**Education (years), OR (95% CI)^a^**					
	0–12	Reference	Reference	Reference	Reference
	13–15	1.1 (0.6–2.2)	1.3 (0.5–3.0)	1.2 (0.8–2.0)	1.2 (0.6–2.3)
	16–	1.4 (0.7–2.8)	1.3 (0.5–3.1)	2.0 (1.3–3.2)^b^	1.9 (1.0–3.6)
**Urban residence^e^, OR (95% CI)^a^**		1.9 (1.0–3.8)	0.9 (0.3–2.7)	0.8 (0.4–1.5)	0.5 (0.2–1.4)
**Health status, OR (95% CI)^a^**					
	Excellent/very good	Reference	Reference	Reference	Reference
	Good	1.0 (0.5–2.1)	2.2 (0.8–6.0)	1.9 (1.2–3.2)^b^	3.8 (1.7–8.7)^b^
	Fair	0.9 (0.4–1.8)	1.5 (0.5–4.2)	1.3 (0.8–2.2)	2.0 (0.8–4.6)
	Poor	3.3 (1.1–9.6)	2.5 (0.5–13.5)	1.8 (0.7–4.5)	1.6 (0.3–8.2)
**Test for goodness-of-fit^f^**		*P* = .99	*P* = .31	*P* = .59	*P* = .31

^a^ Odds ratio (95% confidence interval).

^b^ Confidence interval does not include 1.0.

^c^ Not applicable.

^d^ ¥1000 = about US $10.

^e^ Population of at least 150,000 people.

^f^ Hosmer-Lemeshow goodness-of-fit test.

### Perceived Effects of Internet Use on Health Care

Table 5, Table 6, Table 7, and Table 8 show results of perceived effects of Internet use on health care. More than two-thirds of Internet users strongly agreed or agreed that Internet use “improved my understanding of symptoms, conditions, or treatments in which I was interested” (143/210, 68.1%) and “affected the way I eat or exercise” (134/197, 68.0%), while only 23% thought it “improved my ability to manage my health care needs without visiting a doctor or other health care provider.” More than 60% of respondents obtaining any kind of health-related information felt confident after obtaining this information. Most respondents thought that Internet use had no effect on the number of times they visited health professionals (208/234, 88.9%) or telephoned health professionals (216/232, 93.1%), and most had never told health professionals about information they obtained from the Internet (197/236, 83.5%).

**Table 5:** Perceived effects of Internet use on health care understanding and decisions among Internet users

	n	Agree or strongly agree
Improved my understanding of symptoms, conditions, or treatments in which I was interested	210	143 (68.1%)
Affected the way I eat or exercise	197	134 (68.0%)
Led me to seek care from different doctors or health providers than I otherwise would have	190	41 (22%)
Improved my ability to manage my health care needs without visiting a doctor or other health care provider	188	43 (23%)

**Table 6:** Perceived effects of Internet use on feelings of confidence and anxiety among Internet users

Feeling after obtaining information on...	n	Feeling confident	No effect	Feeling anxious
Diseases you have	158	98 (62%)	52 (33%)	8 (5%)
Diseases you want to prevent	125	77 (62%)	46 (37%)	2 (2%)
Treatment of diseases	167	108 (64.7%)	53 (32%)	6 (4%)
On doctors and health care facilities	99	93 (63%)	50 (34%)	4 (3%)
On peers	147	61 (62%)	34 (34%)	4 (4%)
On a healthy lifestyle, fitness, or nutrition	129	82 (64%)	45 (35%)	2 (2%)

**Table 7:** Perceived effects of Internet use on health-related activities (number of times visited or telephoned a physician or other health provider) among Internet users

Number of times...	n	Increased	No effect	Decreased
Visited a physician or other health provider	234	15 (6%)	208 (88.9%)	11 (5%)
Telephoned a physician or other health provider	232	1 (0%)	216 (93.1%)	15 (7%)

**Table 8:** Perceived effects of Internet use on health-related activities (experiences of telling health professionals about health-related information from the Internet) among Internet users

	n	Have done	Tried, but never done	Never tried
Have told health professionals about health-related information from the Internet	236	39 (17%)	12 (5%)	185 (78.4%)

## Discussion

### Principal Results

This study revealed four principal findings. First, the prevalence of Internet use via personal computer for acquiring health-related information was about one-quarter among those surveyed (23.8%), whereas the prevalence of Internet use via cell phone for this purpose was low (6%). The prevalence of Internet use via personal computer was higher than radio (19.1%), but lower than television (60.1%), newspapers (50.3%), and magazines (34.2%). Second, younger people, people with higher education levels, and people with higher household incomes were more likely to acquire health-related information by accessing the Internet via personal computer. Third, the prevalence of Internet use for health-related communication with health professionals, family, friends, or peers was small. Although cell phones were rarely used for this type of communication in general, 12.3% of respondents used cell phones for contacting family or friends specifically for health-related purposes. Finally, the majority of those using the Internet for health care purposes thought the Internet improved their health-related knowledge and affected their lifestyle attitudes, and felt confident after obtaining health-related information through the Internet. In contrast, less than one-quarter of respondents thought Internet use improved their ability to manage their health or changed their health-related activities. We further discuss these four findings below.

First, we found that the prevalence of Internet use via personal computer for health-related information was lower in Japan (24% in 2007) than in the United States (40% in 2001) [2] and Europe (42% in 2005 and 52% in 2007) [5,13]. On the other hand, the prevalence of using traditional sources of information in Japan, such as television and newspapers, was similar to that in the United States and Europe [3,5]. Although the Internet is increasingly being used as a source of health information [10], consumers still value and use traditional information sources in the United States and Europe [14,15,22]. Therefore, the Japanese general population may also still value traditional sources and not widely use the Internet to obtain health-related information.

 Second, our results regarding characteristics of Internet users were consistent with many preceding studies pointing out that older people, people with lower education levels, and people with lower household incomes reported less frequent access to the Internet [23-28]. Since these people may be unfamiliar with the Internet, these characteristics could result in a digital divide, a barrier to accessing health-related information through the Internet [29-33]. A generation gap in digital knowledge and skills is generally acknowledged [34]. Approximately 70% of people aged 50–64 use the Internet in the United States [26,27], whereas we found that hardly anyone over 50 years of age in Japan accessed the Internet. According to a white paper, the number of Internet users among the older people (over 65 years of age) has increased in recent years (28.1% in 2008, 36.9% in 2009) [35]. It is also suggested that active seniors who actively use the Internet could encourage other seniors to use the Internet. Some studies have proposed that less healthy people moderately use the Internet for health-related information [2,36], although some studies show that people with chronic disease are less likely than healthy people to have access to the Internet [37]. Although less healthy people are more likely to ask health professionals about information they find online [38], people who use the Internet for health purposes are more health oriented than people who do not search the Internet [39]. Therefore, people who use the Internet for health purposes might include both less healthy people, who use the Internet for recovery, and more healthy or health-oriented people, who use it for prevention. The relationship between health status and Internet searching behavior remains controversial.

Third, our results suggest that online communication generally remains uncommon in Japan. For communication with family, friends, or peers, cell phones were more used than personal computers. Cell phones were not used as a tool to acquire information, but as a tool for communication by people of all income levels. This could be because even average people in Japan can have advanced cell phones, which are frequently used for email communication with family or friends. For communication with health professionals, the Internet was less used in Japan than in the Unites States [2].

One reason why online communication generally remains uncommon in Japan might be the lack of systems related to eHealth in Japan. In the reimbursement payment system in Japan, the cost of health professional communications with patients is not reimbursed. In the Japanese context of universal health insurance coverage, treatments covered by insurance are not performed together with treatments not covered by insurance. Most health professionals and medical organizations do not promote this communication. Moreover, the legal system pertaining to personal medical information protection in Japan is not fully developed with regard to eHealth. The Japan Internet Medical Association (JIMA) was founded in 1998 to establish a framework for Internet medical usage [40]. JIMA created the Japanese version of the eHealth code of ethics [41], and has also developed the JIMA trust program. However, only 14 medical organizations obtained the JIMA trust mark, possibly because these ethics codes are self-imposed. Although the Ministry of Health, Labour and Welfare (MHLW) instituted the Guidelines on Security Management for Health Information Systems (first in 2005, fourth in 2010), there is no Act similar to the Health Insurance Portability and Accountability Act or Health Information Technology for Economic and Clinical Health Act in the United States [42]. Health professionals and medical organizations autonomously address privacy and security concerns associated with the electronic transmission of health information. Therefore, a nationwide privacy and security framework for eHealth is required in Japan.

The other reason why online communication generally remains uncommon in Japan could be the absence of a well-developed collaborative relationship among industry, government, and academia in Japan. In the United States, the vast majority of active eHealth services, such as WebMD, have been created by ventures put forth by cooperation and innovation among practitioners, researchers, and private industry [43]. Therefore, more collaborative efforts will be required in Japan. Some websites are being developed in Japan. Medical Information Network Distribution Service, which is operated by the Japan Council for Quality Health Care and funded by MHLW, has provided clinical practice guidelines in Japan on the Internet since 2004 [44-47]. Since the Medical Function Information Providing System was instituted by MHLW in 2007, prefectural governments have obligatorily provided information about the structure and outcomes of medical organizations on the Internet [47,48]. Websites created by nonprofit organizations and private industries, as well as pharmaceutical companies, have received awards for being the most informative health care websites in 2010 [49].

Fourth, our study showed that people tended to use the Internet for obtaining health-related information and felt confident in the information they obtained, which is compatible with many studies [36,50-52]. Obtaining information from the Internet, although it did not apparently change their activities, may encourage users to be confident that their ideas are supported. Nevertheless, our study also showed that few Internet users (6.6% via personal computer and 3.0% via cell phone) used the information for communication with health professionals. The frequency of patient communication with health professionals via the Internet was much lower than the frequency of patient visits to a physician’s office (30.7% in a month) [53]. According to Hesse et al, people tend to go to the Internet first [11] and rarely share the information from the Internet with physicians [3]. They still trust face-to-face contact with physicians as their preferred source of health-related information [11,24,54]. The behavioral discrepancy between searching for information on the Internet and not using this information with health professionals might be due to user trust in health professionals, or to user conflict derived from untrusting health professionals whose attitude and behavior are incompatible with the information from the Internet. This is an important topic regarding communication between health providers and health consumers that should be addressed in the future.

### Public Health Implications

Our findings have public health implications. Our results showed that Internet use of health-related information remains less common in Japan than in other developed countries [28]. Japanese aged 50–64 years, a large segment of the baby boomer generation that is going to require increased access to hospitals, did not access information on the Internet because of the digital divide. As active seniors might lead other seniors to follow their example, it could be important to determine the needs of active seniors regarding Internet use. Our results also suggest a behavioral discrepancy. Inadequate use of information obtained from the Internet might have harmful consequences, such as Internet addiction [55,56] or cyberchondria, which is excessive health anxiety generated from online health searches [57,58]. To address these issues, we believe it is important to improve users’ so-called eHealth literacy, defined as “the ability to seek, find, understand, and appraise health information from electronic sources and apply the knowledge gained to addressing or solving a health problem” [48,59,60]. It is appropriate to use the Internet as a supplement to health services rather than as a replacement [13,61-63], and to share the information with health professionals. There are differences between physicians and patients in health literacy [64], but it is also important that health professionals be mindful of patients’ desire for health information [65] and the Internet presence [52,66]. They should discuss the information offered by patients and guide them to reliable and accurate health websites [52]. For searching websites, standards for eHealth, such as the e-Health Code of Ethics 2.0 [41], could be beneficial for both patients and health professionals. Health professionals, public health professionals, and eHealth developers should work together to educate patients about acquiring health information online and critically appraising it [67-69], and to provide tools for them to navigate to the highest-quality information [38].

### Limitations

This study had some limitations. We acknowledge that the study’s sample size was too small to examine the details of individuals who access the Internet via cell phone. The prevalence of Internet use via cell phone was lower than we had expected. Since this study aimed to measure the prevalence of Internet use for health-related information among the general Japanese population, a further study targeting the subset of Internet users who access the Internet via cell phone is required. We also acknowledge that there are no data about the response rate of respondents. In order to examine the extent of selection bias, we compared some indicative items of this survey with a national representative survey [16]. The distributions of age and sex in this survey were almost equal to those reported for the general Japanese population (see Multimedia Appendix 1 and Multimedia Appendix 2). The proportion of respondents using the Internet more than once a week was 41.5% in this survey and 48.5% in the national representative survey, calculated from data that 70.3% of Internet users (69.0% of respondents) use the Internet more than once a week. The small discrepancy between the national results and our findings can be attributed to the difference in survey methods; the national survey was conducted by mail. Nevertheless, given that the discrepancy was small, our results imply that the respondents to this survey were quasi-representative of the Japanese population. We accept that it is hard to discuss the accuracy of the prevalence of Internet use in Japan since there are no Japanese studies or data for comparison. Although repeated cross-sectional surveys are necessary to determine trends and associations, this study is useful in providing fundamental data in Japan.

### Conclusions

In 2007, Japanese moderately used the Internet via personal computers for health purposes, and rarely used the Internet via cell phones. Older people, people with lower education levels, and people with lower household incomes were less likely to access the Internet via cell phone. The Internet moderately improved user health-related knowledge and attitudes, and encouraged user confidence in health-related information. However, it seldom changed their health-related abilities and activities, and was not often used for communicating with physicians. The paucity of Internet use for communication with physicians might be due to the payment system in Japan. Moreover, Internet users did not generally share the information they obtained from the Internet with health professionals. The health-related information from the Internet was inadequately used. Although cell phones were used as a communication tool for health purposes, the reimbursement payment system in Japan might be an obstacle to communication between health providers and health consumers. To encourage this communication, it is important to improve eHealth literacy, especially in middle-aged people. It is also important to make adequate amendments to the reimbursement payment system and nationwide eHealth privacy and security framework, and to develop a collaborative relationship among industry, government, and academia.

## Multimedia Appendix 1

Characteristics of survey participants and the Japanese population.

## Multimedia Appendix 2

Health status of survey participants and the Japanese population.

## Abbreviations

JIMA: Japan Internet Medical Association
MHLW: Ministry of Health, Labour and Welfare
