# Supplementary material for: Internet Use for Health-Related Information via Personal Computers and Cell Phones in Japan: A Cross-Sectional Population-Based Survey
Source: J Med Internet Res. 2011 Dec 14;13(4):e110. doi: 10.2196/jmir.1796 (PMC3278096; doi:10.2196/jmir.1796)
Supplement: Supplementary file 1 [file jmir_v13i4e110_app1.pdf]

Appendix 1. Characteristics of survey participants and the Japanese population

	Survey participants		Japanese population [a]	
	N [b]	%	N [b]	%
Age				
15-19	75	6.3	6.3	6.1
20-34	285	23.8	24.5	23.7
35-49	295	24.6	25.3	24.5
50-64	324	27.0	27.0	26.1
65-74	169	14.1	14.7	14.3
75-79	52	4.3	5.6	5.4
Sex				
Male	595	49.6	51.1	49.4
Female	605	50.4	52.3	50.6
Total	1200	100.0%	103.4	100.0%

[a] Japanese population estimates in Sept. 2007 from the Statistics Bureau in Japan (http://www.stat.go.jp/english/data/jinsui/2.htm)

[b] Unit: Survey Participants (people), Japanese population (million people)
