# Supplementary material for: Internet Use for Health-Related Information via Personal Computers and Cell Phones in Japan: A Cross-Sectional Population-Based Survey
Source: J Med Internet Res. 2011 Dec 14;13(4):e110. doi: 10.2196/jmir.1796 (PMC3278096; doi:10.2196/jmir.1796)
Supplement: Supplementary file 2 [file jmir_v13i4e110_app2.pdf]

Appendix 2. Health status of survey participants and the Japanese population

	Survey participants		Japanese population
	N [a]	%	%
Health status [b]			
Excellent/very good	248	20.7	19.6
Good	350	29.2	17.4
Fair	520	43.3	49.1
Poor	82	6.8	13.8
Diseases [c]			
Hypertension	178	14.8	12.8
Diabetes or hyperglycemia	69	5.8	5.7
Obesity	109	9.1	9.3
Hyperlipidemia	86	7.2	15.8

[a] Unit: Survey participants (people)

[b] Calculated from the National Health and Nutrition Survey 2007 (http://www.mhlw.go.jp/bunya/kenkou/eiyou09/01.html)

[c] Calculated from the National Health and Nutrition Survey 2006. Among analyzable participants of National Health and Nutrition Survey 2006 (individuals aged ≥15 years, N=7,728), 4,817 people (62.3%) had health checkups in one year, 992 people (12.8%) were diagnosed with hypertension, 443 people (5.7%) with diabetes or hyperglycemia, 722 people (9.3%) with obesity, and 1223 people (15.8%) with hyperlipidemia. (http://www.mhlw.go.jp/bunya/kenkou/eiyou08/)
